# Evaluation of the Impact of the Ripening Stage on the Composition and Antioxidant Properties of Fruits from Organically Grown Tomato (*Solanum lycopersicum* L.) Spanish Varieties

**DOI:** 10.3390/foods13152337

**Published:** 2024-07-25

**Authors:** Ivan Cruz-Chamorro, Guillermo Santos-Sánchez, Franz Martín, María-Soledad Fernández-Pachón, Dámaso Hornero-Méndez, Isabel Cerrillo

**Affiliations:** 1 Departamento de Biología y Geología, Escuela Superior de Ingeniería, Universidad de Almería, 04120 Almería, Spain; icc967@ual.es; 2 Área de Nutrición y Bromatología, Departamento de Biología Molecular e Ingeniería Bioquímica, Universidad Pablo de Olavide, Ctra. Utrera Km 1, 41013 Seville, Spain; fmarber@upo.es (F.M.); icergar@upo.es (I.C.); 3 Instituto de Investigación en Ciencias de la Alimentación, CIAL (CSIC-UAM), 28049 Madrid, Spain; g.santos@csic.es; 4 Departamento de Fitoquímica de los Alimentos, Instituto de la Grasa-CSIC, Campus Universitario Pablo de Olavide, Ctra. Utrera Km 1, 41013 Seville, Spain; hornero@ig.csic.es

**Keywords:** cherry-like tomato, salad tomato, carotenoid, polyphenol, antioxidant activity

## Abstract

Tomato (*Solanum lycopersicum* L.) is a widely cultivated horticultural crop. It belongs to the Solanaceae family and is known for its high concentration of essential nutrients, including vitamins, minerals, and bioactive compounds with antioxidant properties. The Mediterranean countries, including Italy, Spain, and Greece, have a diverse range of tomato landraces. Assessing the nutritional and bioactive composition of different tomato varieties and their ripening stages is crucial to determine their suitability for the market. Therefore, the aim of this study was to investigate the effect of ripening on nutritional composition (including carotenoids and polyphenols content) and antioxidant activities of fruits of three specific tomato varieties grown in Spain: *Josefina* and *Karelya*, which are cherry-like tomatoes, and *Muchamiel*, a type of salad tomato. In addition to evaluating their characteristics and composition (including carotenoids and polyphenol content), the antioxidant activities of these varieties at three different ripening stages were quantified. As expected, the results reveal that, as the tomatoes matured, their antioxidant capacity increased along with higher levels of carotenoids and polyphenols. Interestingly, cherry-like tomatoes showed a higher antioxidant activity than the salad tomatoes. This investigation emphasizes the role of fruit ripening in increasing carotenoid levels, which contribute to the antioxidant activity of three tomato varieties.

## 1. Introduction

Tomato is one of the most popular and widely consumed vegetables in the Mediterranean diet and has therefore been extensively characterized in terms of its nutritional profile and bioactive compounds, being an excellent source of ascorbic acid, carotenoids, and flavonoids [1]. In order to maximize its sensory quality, nutritional composition, and bioactivity, several studies have investigated how different factors, such as the ripening state or tomato variety, influence these aspects [2]. Among the 17 goals of the 2030 Agenda for Sustainable Development, adopted by all United Nations member states in 2015, are (i) responsible production and consumption, (ii) climate action, and (iii) the life of terrestrial ecosystems, with a strong emphasis on reducing greenhouse gas emissions, land use, energy consumption and, to a lesser extent, water use in the cultivation and production of food [3]. Furthermore, organically grown vegetables are a good sustainable alternative, due to the exclusion of the use of synthetic chemicals and respect for the environment [4]. The manner in which tomatoes are grown, as well as the variety of tomato that is grown, can influence the quality of the fruit and may be of significant importance in the production of the crop from a profitable standpoint (obtaining a tomato with optimal quality and long commercial shelf life), reducing costs at the industrial level, and health (obtaining a tomato with a high nutritional density, rich in bioactive compounds, and high antioxidant activity) [5]. Furthermore, organic farming compared to conventional cultivation in terms of quality, nutritional intake, and bioactivity is better [6]. Currently, there are few data on the characterization of organic tomato quality, and the information available to the farmer about the factors that influence it is scarce.

Noncommunicable diseases (NCDs), such as cardiovascular diseases, diabetes, and cancer, killed 41 million people (74% of global deaths) in 2022 [7]. Research supports that oxidative stress, known as an imbalance between reactive oxygen species (ROS) and antioxidant defenses, is part of the triggering and development of the pathophysiological changes associated with noncommunicable diseases [8,9]. In this sense, numerous epidemiological and clinical studies have shown how tomato consumption reduces the risk of contracting numerous diseases, such as cardiovascular diseases or cancer [10]. This beneficial role of tomato consumption has been attributed to its content of polyphenols and carotenoids (mainly lycopene and β-carotene) since these compounds inhibit reactions mediated by ROS. According to numerous studies, the content of these antioxidant compounds in tomato fruits depends on genetic and environmental factors, as well as on the ripening stage of the fruit [11]. In addition, functional foods are gaining attention in the field of nutrition because of their beneficial effects on health and the prevention and treatment of many diseases [12]. In this sense, the extraction of tomato antioxidant compounds, mainly the carotenoid lycopene, for the subsequent generation of functional foods, is gaining attention within the food and pharmaceutical industries [13,14]. Therefore, it is of a great interest to know the variations in the content of antioxidant compounds during the ripening process in order to obtain fruits with the highest bioactivity potential. In light of this well-known evidence presented above, the objective of this study was to evaluate, for the first time, the quality, nutritional, and antioxidant (composition and activity) variations throughout the ripening process of fruits from three Spanish tomato varieties (*Josefina*, *Karelya,* and *Muchamiel*) grown under organic farming practices. The aim was to determine the optimal ripening stage to maximize the nutritional and antioxidant potential of the fruits.

## 2. Materials and Methods

### 2.1. Reagents

Folin and Ciocalteu phenol reagent, gallic acid, Trolox, 2,2-azino-bis-(3-ethylbenzothiazoline-6-sulfonic acid) (ABTS), azo2,20-azobis(2-methylpropionamidine) dihydrochloride (AAPH), sodium fluorescein, 2,2-diphenyl-1-picrylhydrazyl (DPPH), and 2,4,6-Tris(2-pyridyl)-s-triazine (TPTZ) were purchased from Sigma-Aldrich (St. Louis, MO, USA).

### 2.2. Experimental Design and Sample Collection

Bioalverde S.L. (Seville, Spain) supplied the organic tomato samples. Specifically, 30 plants of each variety, *Josefina* and *Karelya*, which are cherry-like tomatoes, and *Muchamiel*, a type of salad tomato, were planted in the greenhouse, divided into 2 different plots (with 15 plants of each species/area).

Three consecutive ripening stages (RS) were selected according to the external color as follows: RS1—changing color fruit, in which green-colored areas are more prevalent than red ones; RS2—changing color fruit, in which red-colored areas are more prevalent than green ones; and RS3—fully mature fruit with an intense red color. A minimum of 40 tomato fruits were collected for each ripening stage and for each variety. Nondestructive determinations (such as fruit size, weight, and color) were carried out in 20 fruits (size and weight) or 10 fruits (color). The same fruits used for the measurement of the color (*n* = 10) were used for the determination of the texture (firmness). For the rest of the measurements, homogenates of tomatoes were used. Specifically, determinations of moisture (water content), pH, and SST were performed immediately after the preparation of the homogenate, while determinations of antioxidant activity, total phenols, and titratable acidity were carried out in frozen aliquots of the homogenate supernatant. The determinations of the carotenoid and nutritional composition were carried out on lyophilized aliquots of the homogenate.

### 2.3. Physicochemical Characterization and Quality Parameters

#### 2.3.1. Fruit Weight and Size

Fruits of each variety were weighed using an analytical balance (model AX224, Sartorius AG, Goettingen, Germany). Fruit sizes were determined as the equatorial diameter and measured with a digital caliper (VWR).

#### 2.3.2. Color

The evaluation of the fruit color was carried out using the colorimetry technique. The color was also expressed in terms of the CIE L* (luminosity, whiteness or brightness/darkness), a* (redness/greenness), and b* (yellowness/blueness) coordinates [15]. Colorimetric measurements on the tomatoes were performed using a BYK-Gadner, model 9000, Color-view™ spectrophotometer (Silver Spring, MD, USA). Results are expressed as a/b.

#### 2.3.3. Total Soluble Solid (TSS) Content

The TSS content was measured in a small sample (2 drops) of the fruit homogenate supernatant using a portable handheld refractometer (model 0-32° BRIX ATC, Bellingham + Stanley, Kent, UK) with an accuracy of ±0.1° Brix [16].

#### 2.3.4. pH Determination

The pH value was determined using a pH meter (model pH 1000L, VWR International Eurolab S.L., Barcelona, Spain) using the homogenate prepared from the collected fruits.

#### 2.3.5. Texture

Texture (firmness) was measured with an Instron Universal Testing Machine (Canton, MA, USA) fitted with a Kramer shear compression cell. The cross-head speed was 200 mm/min. The firmness (shear compression force) of the tomato fruits was expressed as N/100 g, and the value was the mean of ten measurements, each of which was performed on one fruit for cherry-like varieties and with one quarter of the fruit for the *Muchamiel* cultivar.

#### 2.3.6. Proximate Composition 

The proximate composition of the fruits was evaluated for each cultivar at the selected ripening stages (RS1, RS2, and RS3). Dry matter, moisture, ash, protein, fat, and carbohydrate determinations were made using AOAC methods [17], and the results are expressed in relative units (%). The dry matter content and moisture were determined by gravimetry using a moisture balance (model MB35, OAHUS, Barcelona, Spain). The mineral content (ash) was determined by the calcination method. The total protein content was estimated using a LECO Elemental Analyzer (model CHNS-932, Leco Corporation, St. Joseph, MI, USA) for nitrogen determination. The protein content was calculated from the nitrogen content (Dumas method) as follows: Protein (%) = Nitrogen (%) × 6.25 [18]. The total lipid (fat) content was determined using the Soxhlet method [19]. The determination of total carbohydrates (including fiber) was carried out using the differential method by subtracting the sum of percentages of the rest of nutrients (total proteins, total lipids, minerals, and moisture) from 100 [17]. Each parameter was analyzed in triplicate.

#### 2.3.7. Carotenoid Analysis 

Carotenoid pigments were extracted and analyzed using HPLC following the procedure described in de los Santos et al. [20]. Briefly, an aliquot (0.045–0.050 g) of the lyophilized and homogenized sample was placed in a 2 mL micro-centrifuge tube and extracted with 1 mL of acetone-THF (4:1; containing 0.1% BHT) by sonication for 1 min, followed by a vigorous shaking in a mixer for 10 min. The extraction procedure was repeated twice, without the addition of new solvent. The resulting extract was centrifuged at 12,000× *g* for 10 min and 4 °C, and the upper layer stored at −30 °C until analyzed by HPLC. The quantitative analysis of carotenoids was carried out by HPLC according to the method of Mínguez-Mosquera and Hornero-Méndez [21] with minor modifications. The HPLC system consisted of a Waters e2695 Alliance chromatograph equipped with a Waters 2998 photodiode array detector and controlled with Empower2 software Build 2154 (Waters Cromatografía, SA, Barcelona, Spain). A reversed phase C18 column (200 mm × 4.6 mm, 3 μm, Mediterranea SEA18; Teknokroma, Barcelona, Spain), fitted with a guard column of the same material (10 mm × 4.6 mm), was used. Separation was achieved by a binary gradient elution with an initial composition of 75% acetone and 25% deionized water, linearly increased to 95% acetone in 10 min, then held for 7 min, raised to 100% in 3 min, and held constant for 10 min. The initial conditions were reached in 5 min. The column temperature was maintained at 25 °C and the sample compartment was cooled to 15 °C. An injection volume of 10 μL and a flow rate of 1 mL/min were used. Detection was carried out at 450 nm, and online spectra were acquired in the wavelength range of 330 to 650 nm with a resolution of 1.2 nm. Quantification was carried out using external standard calibration curves prepared with previously isolated lycopene and β-carotene standards and purified in our laboratory. Calibration curves were prepared in the range of 0.5–50.0 μg/mL and were constructed by plotting the peak area at 450 nm versus the pigment concentration. All operations were performed under dim light to prevent isomerization and degradation of carotenoids. Each sample was analyzed in triplicate, and a chromatographic analysis was carried out on the same day as the preparation of the extracts. The results are reported as mg/kg dry weight.

#### 2.3.8. Total Polyphenol Determination

The total polyphenols were quantified using the Folin and Ciocalteu method. A 20 μL homogenate sample (diluted 1:2 with distilled water) was mixed with 100 μL Folin and Ciocalteu phenol reagent and with 80 μL 7.5% Na_2_CO_3_ solution. After incubation for 2 h at room temperature and in the dark, the absorbance was read at 765 nm with a Synergy™ HT-multimode microplate reader (Biotek Instruments, Winooski, VT, USA). A calibration curve using different concentrations of gallic acid (25–250 mg/L) was used to calculate the results as gallic acid equivalents (mg/kg dry weight). 

### 2.4. Antioxidant Capacity

All assays were performed using the homogenate of the collected fruits at each stage of ripening.

#### 2.4.1. Ferric Reducing Antioxidant Power (FRAP) Assay

The ferric reducing ability was estimated according to the procedure of Delgado-Andrade et al. [22]. A 20 µL homogenate sample (diluted 1:20 with water) was mixed with 280 µL FRAP solution (0.83 mM TPTZ and 1.66 mM FeCl_3_ × 6H_2_O in 0.25 M acetate buffer, pH 3.6). After 30 min of incubation at 37 °C, the absorbance at 595 nm was measured with the microplate reader. The values were extrapolated by a Trolox standard curve. 

#### 2.4.2. Trolox Equivalent Antioxidant Capacity (TEAC) Assay

The TEAC assay was performed following the procedure described by Delgado-Andrade et al. [22]. To perform the assay, the ABTS radical solution was used. A total of 280 µL of this solution was mixed with 20 µL of homogenate sample (diluted 1:30 with water). After 30 min of incubation at 30 °C, the ABTS radical content was quantified by a microplate reader at 730 nm. The TEAC values were extrapolated using a Trolox standard curve.

#### 2.4.3. Oxygen Radical Absorbance Capacity (ORAC) Assay

The ORAC assay was performed according to Ou et al. [22], with some minor modifications. A 50 µL homogenate sample (diluted 1:500 with phosphate buffer) was added to 100 µL of sodium fluorescein (2.93 µg/µL) and incubated for 15 min at 37 °C. Then, 50 µL AAPH (60.84 mM) was added to generate peroxyl radicals. Therefore, every 5 min, over 2 h, the decay of fluorescein at its maximum emission of 528 nm was measured using the microplate reader. The area under the curve (AUC) was calculated using a Trolox calibration curve, and the data are expressed as millimole Trolox equivalent/L.

#### 2.4.4. 2,2-Diphenyl-1-picrylhydrazyl (DPPH) Radical Scavenging Activity

The DPPH assay was carried out according to a previous study [22]. A 40 µL homogenate sample (diluted 1:10 with distilled water) were added to 200 µL methanol and mixed with 60 µL DPPH radical solution (0.23 mg/mL). After 1 h of incubation at 30 °C, the DPPH radical was measured at 520 nm with a microplate reader. The results were calculated as a percentage of DPPH radical scavenging activity by the following equation:

$$\%DPPH\;radical\;scavenging\;activity=\frac{Abs\;C-Abs\;S}{Abs\;C}\times 100$$

where C is the control group (H_2_O + methanol + DPPH radical solution) and S is the sample.

### 2.5. Statistical Analysis 

The results are shown as the mean ± standard deviation (SD) and a two-way ANOVA test was applied, followed by multiple comparisons and Tukey’s test correction. Correlations between the antioxidant activity assayed by the DPPH radical and the different carotenoids and polyphenol content were analyzed by Pearson’s correlation. Differences with a *p*-value ≤ 0.05 were considered statistically significant. Data were graphed and analyzed with GraphPad Prism 8 (GraphPad Software, San Diego, CA, USA).

## 3. Results

### 3.1. Quality Parameters of Tomato Varieties during the Ripening Process

The fruit quality parameters obtained for the three studied cultivars throughout the ripening process are summarized in Table 1. The cherry-like tomato fruits showed a mean weight of 17 g, which contrasted with the salad tomato, presenting a mean fruit weight of up to 244 g. This observation was also in accordance with the size of the fruit (determined as the equatorial diameter) of 31 mm and 83 mm for the cherry-like and salad tomato fruits, respectively. Interestingly, there was a marked difference in firmness between the two types of tomatoes: the average firmness in cherry-like fruits was double compared to that of the salad tomatoes, 21.4 N/g and 10.6 N/g respectively. Finally, although the two types of tomatoes possessed a mean pH of 4, the TSS of the cherry-like tomatoes was around 6.9 °Brix, while that of the salad tomatoes was 3.8 °Brix. In terms of variations during the ripening process, the weight and size of the fruits varied over time, depending on the variety. Although the *Josefina* variety showed an increase in weight and size during the ripening process, the *Muchamiel* variety reduced its weight and size in RS3. The *Karelya* variety, however, did not show changes in weight and size throughout the process. The pH value also evolved differently depending on the tomato variety. The two cherry varieties showed an increase in pH levels throughout the ripening process, while the pH values in the *Muchamiel* variety did not change.

**Figure 1:**
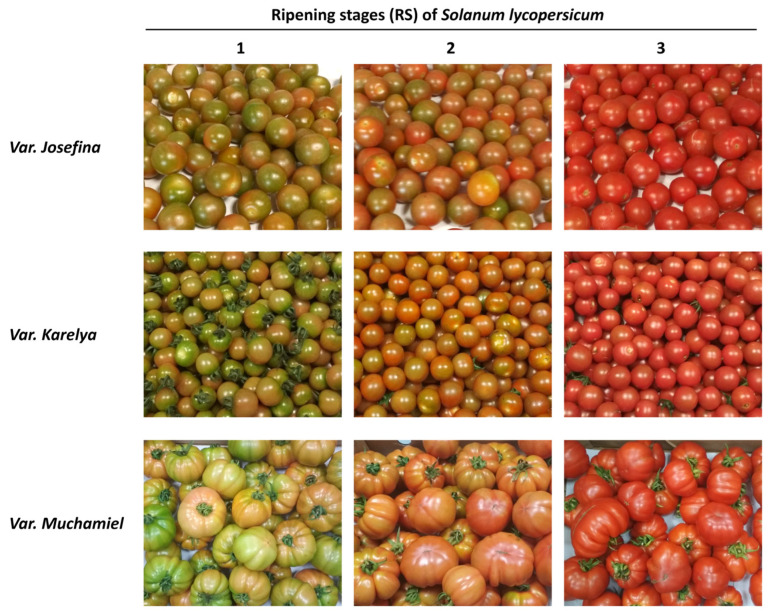
Selected ripening stages (RS), based on fruit color, for the studied cultivars (*Josefina*, *Karelya*, and *Muchamiel*).

The three tomato varieties showed a significant reduction in the firmness values throughout the ripening process, while the TSS and color increased significantly, reaching the highest levels in RS3. The color changes along the ripening process are shown in Figure 1.

### 3.2. Nutritional Composition of Tomato Fruits during the Ripening Process

As shown in Table 2, water was the main constituent of the fruits of the three varieties of tomatoes, with *Muchamiel* being the variety that presented the highest moisture content (95.50 ± 0.84%, value in RS1), which was significantly different from the moisture content of the *Josefina* (RS1: 92.9 ± 0.55%, *p* < 0.0001) and *Karelya* (RS1: 91.9 ± 0.33%, *p* < 0.0001) varieties. Furthermore, carbohydrates were the most representative macronutrients in tomatoes, followed by proteins and lipids. The *Karelya* variety showed significantly higher levels of proteins (0.27 ± 0.02%) and carbohydrates (9.11 ± 0.03%) in RS3 compared to *Josefina* (0.20 ± 0.01%, *p* = 0.029; 7.67 ± 0.19%, *p* < 0.0001, respectively) and *Muchamiel* (0.22 ± 0.03%, *p* = 0.044; 3.91 ± 0.94%, *p =* 0.05, respectively). *Muchamiel* was the variety with the highest levels of lipids (0.19 ± 0.02%). No significant differences in lipid content were observed between *Muchamiel* and *Josefina (p* = 0.166) or *Karelya* (*p* = 0.258). Regarding minerals, the *Karelya* variety had the highest levels in RS3 (0.46 ± 0.02), which was significantly higher than the values of *Muchamiel* (0.34 ± 0.02%, *p* < 0.0001) and *Josefina* (0.24 ± 0.03%, *p* < 0.0001).

Similarly, the nutritional composition of the fruits of each tomato variety varied throughout the ripening process (Table 2). The water content showed a statistically significant reduction in RS3 for both cherry varieties compared to RS1 (*Josefina*: *p* = 0.027; *Karelya*: *p* < 0.0001), while the *Muchamiel* variety did not show changes in the value of the moisture content (RS1 compared to RS3 *p* = 0.789). In terms of macronutrients, the three varieties showed a statistically significant increase (*p* < 0.05) in the lipid content during the ripening process, while the carbohydrate content increased only in the cherry varieties (*Josefina*: *p* = 0.0002; *Karelya*: *p* < 0.0001) but not in the *Muchamiel* variety (*p* > 0.05). Furthermore, *Karelya* was the only variety to show a statistically significant increase (*p* = 0.003) in protein levels during the ripening process. However, the mineral content increased significantly in *Karelya* (*p* = 0.016), without any changes in the other two varieties (*p* > 0.05).

### 3.3. Evolution of Polyphenol Content in Tomato Varieties during the Ripening Process

As shown in Table 3, the three tomato varieties showed an increase in the total polyphenol levels throughout the ripening process. In particular, *Josefina* and *Karelya* varieties showed a significant increase in total polyphenols already in RS2 of the ripening process (*Josefina*: *p* = 0.022; *Karelya*: *p* = 0.003, with respect to RS1), without significant differences between RS2 and RS3. However, the *Muchamiel* variety did not show a significant increase in the levels of the total polyphenol content in RS2 (*p* = 0.725 compared to the RS1 stage), but it increased its polyphenol content in the advanced RS3 stage (compared to RS1, *p* = 0.0001; and RS2, *p* = 0.003). 

### 3.4. Changes in the Carotenoid Levels in Tomato Fruits during the Ripening Process

Variations in total and individual carotenoid contents (total carotenoid, lycopene, and β-carotene) were evaluated (Table 3). Therefore, the fruits of the three tomato varieties showed a significant increase (*p* < 0.0001) in total carotenoids during the ripening process, reaching the highest levels in RS3 (Table 3). In more detail, the ripening process allowed for a significant increase (*p* < 0.0001) in lycopene levels in all three varieties, reaching the highest levels in the RS3 stage (236 ± 18.60 mg/kg dry weight, 245 ± 22.30 mg/kg dry weight, and 392 ± 28.60 mg/kg dry weight, respectively). On the other hand, β-carotene levels increased differently. While the *Muchamiel* variety reached its highest β-carotene levels in RS3 (59.50 ± 12.20) (*p* < 0.0001), the *Josefina* and *Karelya* varieties reached the highest levels in RS2 and RS3, respectively (*Josefina*: 74.30 ± 3.84; *p* < 0.0001; *Karelya*: 48.30 ± 3.75 *p* = 0.0003), without observed differences between stages RS2 and RS3 (*Josefina*: *p* = 0.968; *Karelya*: *p* = 0.507).

### 3.5. Effect of Ripening Process on Antioxidant Capacity of Tomato Varieties

To find out if the antioxidant capacity of tomatoes increased during the ripening process, the FRAP, ORAC, TEAC, and DPPH antioxidant tests were carried out at each ripening stage.

**Figure 2:**
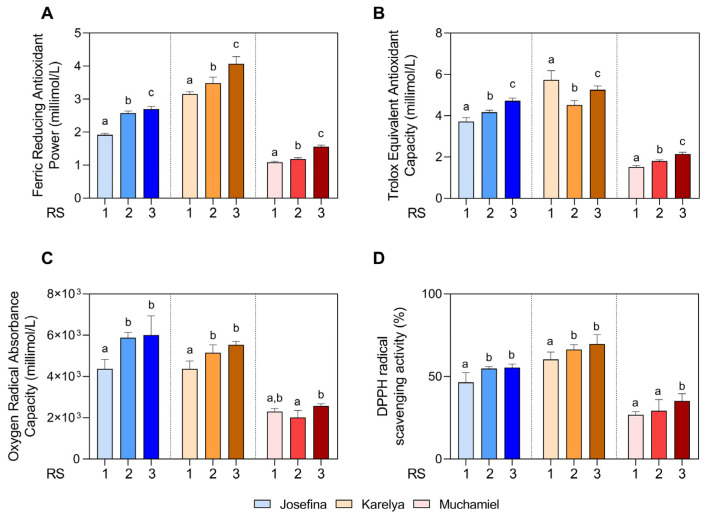
Antioxidant activity in the three tomato varieties according to ripening stage using different assays: (**A**) Ferric Reducing Antioxidant Power. (**B**) Trolox Equivalent Antioxidant Capacity. (**C**) Oxygen Radical Absorbance Capacity. (**D**) DPPH (2,2-diphenyl-1-picrylhydrazyl). RS, ripening stage. Different letters represent a statistical difference (*p* ≤ 0.05) within each variety.

As observed in Figure 2A, the FRAP value increased in the RS2 and RS3 stages, reaching the maximum value in RS3 in the three tomato varieties studied (RS3: *Josefina*: 2.70 ± 0.08 mmol/L; *Karelya*: 4.07 ± 0.22 mmol/L; and *Muchamiel*: 1.56 ± 0.05 mmol/L, *p* < 0.0001 with respect to RS1). As shown in Figure 2B, the capacity to scavenge the ABTS radical (TEAC assay) increased throughout the ripening process for the *Josefina* (RS1: 3.72 ± 0.19 mmol/L; RS2: 4.17 ± 0.10 mmol/L; RS3: 4.72 ± 0.13 mmol/L) and *Muchamiel* (RS1: 1.52 ± 0.07 mmol/L; RS2: 1.81 ± 0.06 mmol/L; RS3: 2.14 ± 0.09 mmol/L) varieties. However, the ABTS scavenging capacity decreased significantly in the *Karelya* variety (RS1: 5.74 ± 0.44 mmol/L; RS2: 4.52 ± 0.21 mmol/L; RS3: 5.26 ± 0.19 mmol/L). The results of the ORAC assay show how the oxygen radical absorption capacity increased in RS2 (*Josefina*: *p* = 0.002; *Karelya*: *p* = 0.005) and RS3 (*Josefina*: *p* = 0.001; *Karelya*: *p* = 0.0002) for the cherry varieties, without significant differences (*Josefina*: *p* = 0.934; *Karelya*: *p* = 0.194) between these stages (Figure 2C). However, the *Muchamiel* variety did not show significant differences in ORAC at any ripening stage (Figure 2C).

Regarding DPPH radical scavenging activity, the varieties *Josefina* and *Karelya* increased their activity in stages RS2 (*Josefina*: 54.8 ± 1.22%, *p* = 0.006; *Karelya*: 66.4 ± 2.93%, *p* = 0.03 with respect to RS1) and RS3 (*Josefina*: 55.4 ± 2.15%, *p* = 0.005; *Karelya*: 69.7 ± 5.69%, *p* = 0.001 with respect to RS1), without significant differences were found between them (Figure 2D). However, the *Muchamiel* variety significantly (*p* = 0.0009) increased the DPPH radical scavenging capacity in RS3 (35.2 ± 4.35%), with no significant differences being found between stages RS1 (26.8 ± 1.90%) and RS2 (29.3 ± 6.72) (Figure 2D). 

Regarding the differences in the antioxidant capacity between varieties, both cherry tomatoes stood out for their higher antioxidant activity compared to the *Muchamiel* salad tomato. Among the cherry varieties, *Karelya* showed the highest antioxidant activity in all assays, except for ORAC.

### 3.6. Correlation between Bioactive Compounds and Antioxidant Activity

To identify a possible relationship between the carotenoid or polyphenol content and the antioxidant capacity, the data were analyzed using Pearson’s correlation. As shown in Table 4, in the *Josefina* variety, significant and positive correlations were observed between the levels of total carotenoids, β-carotene, and lycopene with the values of FRAP, TEAC, and ORAC, as well as negative correlations between these and DPPH.

In contrast, the total polyphenols showed positive correlations with FRAP and TEAC values, but no statistically significant correlation was observed with ORAC and DPPH. In the *Karelya* variety (Table 5), the total carotenoids, β-carotene, and lycopene showed positive and significant correlations with FRAP and ORAC values and negative correlations with DPPH values. However, the carotenoid levels did not show a significant correlation with TEAC.

In the case of polyphenols, positive and significant correlations were observed with FRAP and ORAC, but not with DPPH and ABTS. For the *Muchamiel* variety (Table 6), positive and significant correlations were observed between total polyphenols, total carotenoids, β-carotene, and lycopene and FRAP and TEAC. Regarding the other assays, a positive correlation was observed between β-carotene and ORAC values, while negative correlations were found between total carotenoid and lycopene levels and DPPH radical values.

## 4. Discussion

This study evaluated the quality, nutritional, and antioxidant variations during the ripening process of three Spanish tomato varieties (including two cherry-like and one salad tomatoes) were evaluated. Tomatoes were grown under organic farming conditions and evaluated at three different stages of ripening, according to the color of the fruit. The ripening process was shown to affect each tomato variety differently in terms of quality parameters, nutritional properties, and antioxidant activities. 

First, the quality of each tomato fruit was assessed by analyzing the pH, TSS, and moisture content. The pH values, which determine the acidity of the fruit, were similar to those observed in other studies [23,24]. The TSS is the index that most influences the production performance of fruits for industrial processing. This depends on the variety and the agronomic conditions, including the irrigation and ripening period. In this sense, tomato fruits grown under organic conditions have been observed to have higher TSS values (3.90 °Brix) compared to those fruits grown under conventional conditions (3.21 °Brix) [6]. The tomatoes in our study showed an increase in TSS throughout the ripening process. However, the observed levels were comparable to those previously documented for other tomato varieties [23,25,26], regardless of the cultivation method employed (organic or conventional). Finally, it was found that the content of dry matter, which is essential for the quality of the fruit and has a positive effect on the organoleptic characteristics of the product, is within the normal range from 5 to 7.5% [27]. The rest of the quality parameters, including weight, size, color, and firmness, showed values similar to those previously described in the same or others tomato varieties [28,29], except the weight of the *Karelya* and *Muchamiel* varieties, which were higher than those established by other authors [30,31].

In terms of nutritional analysis, the levels of carbohydrates, lipids, and minerals were similar to those of other previously characterized tomato cultivars [32,33,34]. However, the protein fraction was slightly below average levels, probably due to the tomato varieties themselves and/or the effect of cultivation under organic conditions. Additionally, the ripening process did not affect the nutritional composition of the *Muchamiel* variety, which only showed a slight increase in lipid fraction in RS3. However, cherry varieties, in addition to showing a decrease in moisture in RS2, showed an increase in protein, carbohydrate, and mineral contents, in some cases (such as carbohydrates) already in the RS2 stage.

Polyphenol and carotenoids levels increased significantly during the ripening process. This is of great interest as the functional value of tomatoes is mainly determined by the content of carotenoids and polyphenols, which have attracted the interest of many researchers in the field because of their biological and physicochemical properties. Lycopene is the main carotenoid in tomatoes and is responsible for their characteristic red color. This bioactive component, along with other carotenoids, has been shown to inhibit cancer cell proliferation, as well as exert a powerful antioxidant effect, of great importance in counteracting the development of numerous diseases, such as cardiovascular disease and those related to age [35,36,37,38]. In this sense, the carotenoid content has been shown to depend on numerous factors such as the stage of ripening, the variety of tomato, or the cultivation conditions. With regard to this last factor, the lycopene content of tomatoes grown under organic conditions has been shown to be higher than those grown under conventional practices [6]. In our study, the levels of lycopene, β-carotene, and total carotenoids in the ripening stage RS3 were in agreement with those of previous studies [1,15,24,26,39,40].

Furthermore, total polyphenol levels were comparable to those observed in previous studies [40,41,42] using conventional cultivation conditions. Our results show that tomato ripening significantly increases the levels of bioactive compounds, thus enhancing the functional value of tomatoes. Our results can also indicate the optimal harvesting time of tomatoes according to the bioactive compound of interest to optimize its content (Table 7). 

To evaluate the antioxidant activity of tomatoes during the ripening process, four antioxidant assays were used: FRAP, to measure the total antioxidant status, and ORAC, TEAC, and DPPH to evaluate the radical scavenging capacity. The correlation analysis showed how the exerted antioxidant effect depends on each bioactive compound and the different types of antioxidant assays used. In fact, due to the chemical nature of the method used to measure the antioxidant activity, it is important to use several methods to evaluate a possible effect mediated by different mechanisms of action. While the TEAC and DPPH assays measure the antioxidant capacity exerted by water-soluble (polyphenols) and lipid-soluble (carotenoids) compounds, the ORAC and FRAP assays can only measure those that are water-soluble. Our results show that the global antioxidant status increased significantly during the ripening process, reaching the highest levels in RS3. Although other authors have studied the antioxidant capacity of several tomato varieties [15,24,33,40,43], this is the first time that the antioxidant activity was evaluated in the *Karelya*, *Josefina*, and *Muchamiel* varieties at three different stages of ripening and grown under organic conditions. The total antioxidant capacity has been shown to be higher in tomatoes that have been grown under organic conditions compared to those grown following conventional practices [34,44]. This is consistent with the findings of Vinha et al. [6], who reported DPPH inhibition values in tomatoes grown under organic practices (62.1 ± 1.3%) similar to those observed in the present study. Both values were higher than those observed for tomatoes grown using conventional cultivation methods (58.4 ± 0.7%). 

Considering the optimal time to harvest the fruit based on its maximum antioxidant capacity, harvesting should be conducted when the tomatoes are fully ripe (stage RS3). However, in some cases, harvesting could be brought forward depending on whether the final goal is to obtain the greatest scavenging activity. In this sense, cherry tomatoes reached their highest scavenging activity in RS2, while salad tomatoes had their highest values of DPPH, ORAC, and TEAC in RS3. Acquiring this knowledge would lead to optimizing the antioxidant activity and scavenging capacity of tomatoes according to the variety.

## 5. Conclusions

This study examines the various effects of ripening on different tomato varieties, including their impact on quality, nutritional content, and antioxidant levels. As expected, ripening significantly improves the levels of carotenoids and polyphenols in the varieties studied, thus improving the functional value of tomatoes and their antioxidant activity, especially in fully ripe tomatoes. Harvesting tomatoes during ripening stage 3 maximizes the bioactive content and antioxidant capacity in salad tomatoes, but not in cherry tomato varieties. In particular, the TEAC, ORAC, and DPPH assays showed a higher antioxidant capacity in RS2 in the *Josefina* and *Karelya* varieties. The results obtained in this study provide useful information on the optimal ripening times for the tomato varieties studied, which can be used to guarantee their quality even before harvest.

## Figures and Tables

**Table 1:** Changes in the quality parameters of tomato fruits cultivars *Josefina*, *Karelya*, and *Muchamiel*, at three different ripening stages.

	*Josefina*			*Karelya*			*Muchamiel*		
	RS1	RS2	RS3	RS1	RS2	RS3	RS1	RS2	RS3
Weight (g)	15.1 ± 2.81 ^a^	16.5 ± 3.21 ^b^	20.6 ± 4.68 ^c^	17.3 ± 2.49 ^a^	17.9 ± 1.94 ^a^	18.2 ± 2.80 ^a^	254 ± 74.10 ^a,b^	258 ± 88.00 ^a^	225 ± 79.50 ^b^
Size (equatorial diameter, mm)	30.2 ± 2.10 ^a^	29.3 ± 1.92 ^b^	33.0 ± 2.78 ^c^	31.4 ± 2.16 ^a^	31.6 ± 1.62 ^a^	31.3 ± 1.68 ^a^	84.2 ± 10.20 ^a^	84.6 ± 12.40 ^a^	80.9 ± 12.10 ^b^
Firmness (N/g)	27.4 ± 2.32 ^a^	24.9 ± 4.11 ^a^	14.5 ± 6.38 ^b^	25.3 ± 3.75 ^a^	19.2 ± 2.91 ^b^	17.0 ± 1.64 ^b^	15.0 ± 3.88 ^a^	10.0 ± 1.75 ^b^	6.74 ± 1.57 ^c^
pH	3.88 ± 0.01 ^a^	3.92 ± 0.01 ^b^	4.07 ± 0.01 ^c^	3.88 ± 0.09 ^a^	3.91 ± 0.03 ^a^	4.09 ± 0.08 ^b^	4.23 ± 0.06 ^a^	4.21 ± 0.03 ^a^	4.23 ± 0.05 ^a^
TSS (°Brix)	5.97 ± 0.10 ^a^	6.70 ± 0.06 ^b^	7.37 ± 0.08 ^c^	6.25 ± 0.05 ^a^	6.75 ± 0.05 ^b^	8.10 ± 0.09 ^c^	3.49 ± 0.14 ^a^	3.80 ± 0.12 ^b^	4.03 ± 0.09 ^c^
Color (a/b)	0.07 ± 0.13 ^a^	0.32 ± 0.13 ^b^	0.75 ± 0.10 ^c^	0.16 ± 0.10 ^a^	0.45 ± 0.10 ^b^	0.79 ± 0.16 ^c^	0.28 ± 0.15 ^a^	0.59 ± 0.14 ^b^	0.81 ± 0.13 ^c^

RS, ripening stages. For each parameter and each cultivar, different letters indicate a statistically significant difference (*p* ≤ 0.05) among ripening stages.

**Table 2:** Proximate composition of tomato fruits (cultivars *Josefina*, *Karelya*, and *Muchamiel*) at three different ripening stages.

	*Josefina*			*Karelya*			*Muchamiel*		
	RS1	RS2	RS3	RS1	RS2	RS3	RS1	RS2	RS3
Moisture (%)	92.90 ± 0.55 ^a^	92.40 ± 0.51 ^a,b^	91.10 ± 1.68 ^b^	91.90 ± 0.33 ^a^	91.50 ± 0.12 ^b^	90.00 ± 0.21 ^c^	95.50 ± 0.84 ^a^	95.70 ± 0.36 ^a^	95.30 ± 0.77 ^a^
Proteins (%)	0.19 ± 0.02 ^a^	0.18 ± 0.02 ^a^	0.20 ± 0.01 ^a^	0.20 ± 0.01 ^a^	0.23 ± 0.01 ^a^	0.27 ± 0.02 ^b^	0.20 ± 0.02 ^a^	0.19 ± 0.02 ^a^	0.22 ± 0.03 ^a^
Lipids (%)	0.10 ± 0.02 ^a^	0.13 ± 0.02 ^a^	0.16 ± 0.02 ^b^	0.11 ± 0.02 ^a^	0.12 ± 0.01 ^a^	0.17 ± 0.03 ^b^	0.15 ± 0.04 ^a^	0.14 ± 0.02 ^a^	0.19 ± 0.02 ^b^
Carbohydrates (%)	6.54 ± 0.10 ^a^	7.29 ± 0.15 ^b^	7.67 ± 0.19 ^b^	7.40 ± 0.17 ^a^	7.89 ± 0.09 ^b^	9.11 ± 0.01 ^c^	3.70 ± 1.00 ^a^	3.56 ± 0.47 ^a^	3.91 ± 0.94 ^a^
Minerals (%)	0.23 ± 0.02 ^a^	0.22 ± 0.03 ^a^	0.24 ± 0.03 ^a^	0.38 ± 0.02 ^a^	0.40 ± 0.03 ^a^	0.46 ± 0.02 ^b^	0.35 ± 0.08 ^a^	0.33 ± 0.02 ^a^	0.34 ± 0.02 ^a^

RS, ripening stages. For each parameter and each cultivar, different letters indicate a statistically significant difference (*p* ≤ 0.05) among ripening stages.

**Table 3:** Polyphenol and carotenoid content of the *Josefina*, *Karelya*, and *Muchamiel* tomato varieties at the different ripening status.

	*Josefina*			*Karelya*			*Muchamiel*		
	RS1	RS2	RS3	RS1	RS2	RS3	RS1	RS2	RS3
Total polyphenols (mg/kg dry weight)	106.75 ± 12.38 ^a^	118.38 ± 4.71 ^b^	120.04 ± 7.50 ^b^	84.86 ± 5.05 ^a^	101.59 ± 4.07 ^b^	109.56 ± 9.58 ^b^	60.10 ± 3.37 ^a^	60.99 ± 1.87 ^a^	65.24 ± 2.98 ^b^
Total carotenoids (mg/kg dry weight)	97.80 ± 9.10 ^a^	178.00 ± 9.11 ^b^	310.00 ± 23.90 ^c^	82.70 ± 3.78 ^a^	135.00 ± 8.52 ^b^	294.00 ± 26.00 ^c^	133.00 ± 9.62 ^a^	271.00 ± 23.20 ^b^	451.00 ± 28.90 ^c^
Lycopene (mg/kg dry weight)	42.30 ± 3.77 ^a^	103.00 ± 5.62 ^b^	236.00 ± 18.60 ^c^	41.40 ± 2.27 ^a^	88.00 ± 5.43 ^b^	245.00 ± 22.30 ^c^	90.00 ± 7.69 ^a^	220.00 ± 19.10 ^b^	392.00 ± 28.60 ^c^
β-Carotene (mg/kg dry weight)	55.50 ± 5.56 ^a^	74.30 ± 3.84 ^b^	73.80 ± 5.38 ^b^	41.40 ± 1.61 ^a^	46.90 ± 3.32 ^b^	48.30 ± 3.75 ^b^	42.90 ± 2.87 ^a^	51.10 ± 9.19 ^b^	59.50 ± 12.20 ^c^

For each parameter and each cultivar, different letters indicate a statistically significant difference (*p* ≤ 0.05) among ripening stages (RS).

**Table 4:** Pearson correlations between antioxidant activity and carotenoid and polyphenol contents in tomato fruits from the *Josefina* variety.

	Total Polyphenols		Total Carotenoids		β-Carotene		Lycopene	
	r^2^	*p*-Value	r^2^	*p*-Value	r^2^	*p*-Value	r^2^	*p*-Value
vs. FRAP	0.541	**0.020**	0.865	**<0.0001**	0.899	**<0.0001**	0.828	**<0.0001**
vs. TEAC	0.469	**0.049**	0.959	**<0.0001**	0.718	**0.0008**	0.952	**<0.0001**
vs. ORAC	0.271	0.277	0.640	**0.004**	0.633	**0.005**	0.616	**0.006**
vs. DPPH radical	−0.048	0.855	−0.521	**0.026**	−0.528	**0.029**	−0.515	**0.029**

DPPH, 2,2-Diphenyl-1-picrylhydrazyl radical; FRAP, ferric reducing antioxidant power; ORAC, oxygen radical absorbance capacity; r^2^, Pearson correlation coefficient; TEAC, Trolox equivalent antioxidant capacity. The bold number indicates a statistically significant difference.

**Table 5:** Pearson correlations between antioxidant activity and carotenoid and polyphenol contents in tomato fruits from the *Karelya* variety.

	Total Polyphenols		Total Carotenoids		β-Carotene		Lycopene	
	r^2^	*p*-Value	r^2^	*p*-Value	r^2^	*p*-Value	r^2^	*p*-Value
vs. FRAP	0.711	**0.0009**	0.921	**<0.0001**	0.585	**0.017**	0.922	**<0.0001**
vs. TEAC	−0.431	0.074	−0.112	0.659	−0.47	0.064	−0.085	0.736
vs. ORAC	0.718	**0.002**	0.782	**0.0003**	0.669	**0.005**	0.769	**0.0005**
vs. DPPH radical	−0.377	0.123	−0.576	**0.012**	−0.518	**0.028**	−0.560	**0.016**

DPPH, 2,2-Diphenyl-1-picrylhydrazyl radical; FRAP, ferric reducing antioxidant power; ORAC, oxygen radical absorbance capacity; r^2^, Pearson correlation coefficient; TEAC, Trolox equivalent antioxidant capacity. The bold number indicates a statistically significant difference.

**Table 6:** Pearson correlations between antioxidant activity and carotenoid and polyphenol contents in tomato fruits from the *Muchamiel* variety.

	Total Polyphenols		Total Carotenoids		β-Carotene		Lycopene	
	r^2^	*p*-Value	r^2^	*p*-Value	r^2^	*p*-Value	r^2^	*p*-Value
vs. FRAP	0.674	**0.002**	0.950	**<0.0001**	0.935	**<0.0001**	0.939	**<0.0001**
vs. TEAC	0.595	**0.019**	0.955	**<0.0001**	0.819	**0.0002**	0.957	**<0.0001**
vs. ORAC	0.495	0.072	0.410	0.145	0.598	**0.024**	0.384	0.175
vs. DPPH radical	0.225	0.186	−0.387	**0.032**	−0.119	0.489	−0.391	**0.030**

DPPH, 2,2-Diphenyl-1-picrylhydrazyl radical; FRAP, ferric reducing antioxidant power; ORAC, oxygen radical absorbance capacity; r^2^, Pearson correlation coefficient; TEAC, Trolox equivalent antioxidant capacity. The bold number indicates a statistically significant difference.

**Table 7:** Schematic summary of the ripening stages of the three varieties in which the tomatoes could be harvested in order to obtain a specific characteristic (more antioxidant, with more polyphenols, etc.).

Bioactive Compounds	Tomato Varieties		
	Cherry-like		Salad
	*Josefina*	*Karelya*	*Muchamiel*
Total polyphenol index	RS3	RS3	RS3
Total carotenoids	RS3	RS3	RS3
Lycopene	RS3	RS3	RS3
β-Carotene	RS2	RS3	RS3
**Antioxidant capacity**			
Total antioxidant activity (FRAP)	RS3	RS3	RS3
Radical scavenging capacity (TEAC, ORAC, DPPH)	RS2	RS2	RS3

DPPH, 2,2-Diphenyl-1-picrylhydrazyl radical; FRAP, ferric reducing antioxidant power; ORAC, oxygen radical absorbance capacity; RS, ripening status, TEAC, Trolox equivalent antioxidant capacity.

## Data Availability

The original contributions presented in the study are included in the article, further inquiries can be directed to the corresponding author.
